# Transfusion safety in transfusion services: health professionals’ knowledge

**DOI:** 10.1590/0034-7167-2025-0470

**Published:** 2026-08-21

**Authors:** Maíra Cássia Borges de Oliveira, Rosana Amora Ascari, Everaldo José Schörner, Leticia de Lima Trindade, Daiana de Mattia

**Affiliations:** I Universidade do Estado de Santa Catarina. Chapecó, Santa Catarina, Brazil; II Universidade Federal de Santa Catarina. Florianópolis, Santa Catarina, Brazil

**Keywords:** Hemotherapy Service, Blood Transfusion, Blood Safety, Health Personnel, Nursing., Servicio de Hemoterapia, Transfusión Sanguínea, Seguridad de la Sangre, Personal de Salud, Enfermería.

## Abstract

**Objectives:**

to identify health professionals’ knowledge of best practices in blood component transfusion within a blood center network and to encourage critical discussion to inform the development of a minimum framework for a transfusion safety checklist.

**Methods:**

this cross sectional, exploratory, descriptive study included 11 professionals working in the eight hospital-based transfusion services managed by the Santa Catarina Hematology and Hemotherapy Center.

**Results:**

data were collected in July 2025 using a structured questionnaire that addressed professionals’ sociodemographic characteristics and transfusion safety issues within the Santa Catarina blood center network.

**Final Considerations:**

health professionals’ knowledge of best practices in blood component transfusion is essential to ensuring safe and effective care. By highlighting professionals’ perceptions of their day-to-day work in transfusion services, the study encouraged reflection on routine professional practice.

## RESUMEN

**Objetivos:**

identificar el conocimiento de los profesionales de la salud de una red de hemoterapia sobre las buenas prácticas en la transfusión de hemocomponentes y promover una discusión crítica orientada a definir la estructura mínima de una lista de verificación de seguridad transfusional.

**Métodos:**

estudio transversal, exploratorio y descriptivo realizado con 11 profesionales de las ocho agencias transfusionales gestionadas por el Centro de Hematología y Hemoterapia de Santa Catarina.

**Resultados:**

la recolección de datos se realizó en julio de 2025 mediante un cuestionario estructurado con preguntas sobre el perfil sociodemográfico de los profesionales y la seguridad transfusional en la red.

**Consideraciones Finales:**

el conocimiento de las buenas prácticas en la transfusión de hemocomponentes es esencial para garantizar una atención segura y eficaz. Al destacar las percepciones de los profesionales sobre su experiencia laboral en servicios de hemoterapia, el estudio favoreció la reflexión sobre la práctica profesional cotidiana.

## INTRODUCTION

In Brazil, blood components are produced from donated whole blood or through apheresis donations from volunteer donors, as established by national legislation and technical regulations issued by the Ministry of Health. All blood donations must be voluntary and altruistic, with no direct or indirect compensation, while ensuring donor anonymity^(1)^.

Blood component transfusion is a modern therapeutic procedure used to replace blood components depleted by acute or chronic losses, such as those resulting from trauma, major surgery, and oncologic and hematologic diseases^(2,3)^.

In blood component collection, apheresis is an automated procedure in which the donor’s whole blood is processed using specialized equipment to separate and selectively collect components such as platelets, plasma, red blood cells, and granulocytes, while the remaining elements are returned to the donor. Compared with conventional whole-blood donation, apheresis yields larger volumes and purer blood components and enhances safety for both donors and recipients by reducing exposure to multiple donors and lowering the risk of adverse reactions^(4)^.

Current processing techniques allow different blood components to be obtained and stored under conditions that preserve their therapeutic properties. This enables recipients to receive only the components they need, in smaller volumes, thereby reducing the risks associated with transfusion therapy^(5)^. Thus, a single donation can benefit several patients more safely and effectively. In this context, donor screening tests are performed on all donations to ensure donor and recipient safety; these include testing for transfusion-transmissible infectious agents, immunohematologic testing, and screening for hemoglobin S to identify the variant associated with sickle cell disease^(6,7)^.

The decision to transfuse should be based on each recipient’s clinical condition, not solely on laboratory test results. Despite advances in donor recruitment, screening, blood processing, and transfusion safety, this therapy still carries risks inherent in the process itself, including immunosuppression, alloimmunization, and preventable adverse events^(8)^.

Therefore, because transfusion is not risk-free, it should be used only when no other indicated treatment option is available. This decision should be shared with patients and/or their family members so that the risks can be discussed and any questions addressed; transfusion is recommended when its benefits outweigh its risks^(1)^. In contexts such as severe anemia, acute hemorrhage, or hemorrhagic shock, blood component transfusion offers immediate benefits, including restoration of oxygen-carrying capacity, hemodynamic stabilization, and reduced mortality. Although transfusion-related risks remain, restrictive transfusion strategies based on well-defined criteria have been associated with improved clinical outcomes, fewer complications, and shorter hospital stays^(9,10)^.

Health professionals’ knowledge and skills are essential to developing and improving safe blood component transfusion practices and achieving favorable clinical outcomes, especially through team training and capacity-building efforts designed to prevent adverse events^(11)^. Resolution No. 709 of 2022^(12)^ of the Federal Nursing Council (Cofen) emphasizes that nursing technicians and nurses should be properly trained to perform procedures such as collection, storage, quality control, and care for donors and patients, thereby ensuring competent, effective, and safe care^(12)^. In addition to nursing professionals, physicians, pharmacists, biomedical scientists, and biologists also work in transfusion services and are responsible for pretransfusion testing, selecting blood components according to each recipient’s needs, releasing results, and resolving discrepancies^(13)^.

In the state of Santa Catarina (SC), activities across the blood supply chain are coordinated by the Santa Catarina Hematology and Hemotherapy Center (HEMOSC), which accounts for 99% of transfusion medicine coverage statewide, including donor recruitment and voluntary blood and bone marrow donation^(14,15)^. The institution ensures quality control in blood collection, donor screening, blood component production, storage, and distribution to public and private services, while also managing the state blood inventory^(14,15)^.

Against this backdrop, this study addresses the need to identify health professionals’ knowledge of best practices in blood component transfusion as part of efforts to strengthen transfusion safety. In the future, it may support the development of a management tool to assist health professionals working in transfusion medicine in strengthening transfusion safety. This checklist-based tool is expected to make a meaningful contribution to patient safety and the quality of care by helping minimize adverse events and advance the Sustainable Development Goals (SDGs), especially SDG 3 (Good Health and Well-Being) through the promotion of safe, high-quality care; SDG 4 (Quality Education) by fostering professional training; and SDG 16 (Peace, Justice and Strong Institutions) by strengthening ethical and responsible management in health services^(16)^. This research is part of a larger project to develop a checklist-based management tool for implementation across the Santa Catarina blood center network. It addresses the first of four proposed stages: a situational diagnosis of the service under study.

## OBJECTIVES

To identify health professionals’ knowledge of best practices in blood component transfusion within a blood center network and to encourage critical discussion to support the development of a minimum framework for a transfusion safety checklist.

## METHODS

### Study design

This was a cross-sectional, exploratory, descriptive study with a quantitative approach, conducted in July 2025 and reported according to STROBE criteria^(17)^.

### Setting

The study was conducted across the eight hospital-based transfusion services managed by the Santa Catarina Hematology and Hemotherapy Center (HEMOSC), located throughout the state of Santa Catarina: Hospital Regional de São José (São José), Hospital Governador Celso Ramos (Florianópolis), Hospital Florianópolis (Florianópolis), Hospital Infantil Joana de Gusmão (Florianópolis), Hospital Regional do Oeste (Chapecó), Hospital Hans Dieter Schmidt (Joinville), Maternidade Tereza Ramos (Lages), and Hospital Waldomiro Colautti (Ibirama)^(15)^.

The study was conducted in accordance with the ethical guidelines established by national and international regulatory bodies and was approved by the Research Ethics Committees of the State University of Santa Catarina and the Santa Catarina State Health Department. All participants signed the Informed Consent Form.

Participants were approached by email through the blood center network’s care coordination team. The message included a cover letter to be forwarded to potential participants, outlining the study objectives and providing instructions about participation, including a link to the Informed Consent Form and, once consent was provided online, access to the data collection instrument. Potential participants were given 15 days to respond, with weekly reminders sent to extend the response period, limited to two reminders. If no response was received, the invitation was sent to the next eligible professional.

### Participants

The sample was selected purposively and comprised of health professionals with undergraduate degree nurses, biomedical scientists, biochemists, and biologists-working in hospital-based transfusion services across the Santa Catarina blood center network. Professionals who were away from work, on leave, or on vacation were excluded.

### Data source

The data collection instrument consisted of two sections. The first gathered participants’ sociodemographic data and professional experience, and the second focused on transfusion safety in the Santa Catarina blood center network. The instrument included 20 questions, some open-ended and some closed ended, developed by the researchers based on a literature review and professional practice. It was made available online through Google Forms^®^.

Because the study aimed to develop a management tool, the same questionnaire also included items based on the SWOT framework-Strengths, Weaknesses, Opportunities, and Threats-to identify strengths that should be maintained and weaknesses that need to be addressed^(18,19)^. SWOT is a widely used management tool that supports strategic and organizational planning.

### Study size

The eligible sample comprised 16 professionals, as each of the eight hospital-based transfusion services in the Santa Catarina blood center network had two professionals with undergraduate degrees.

### Statistical methods

After collecting through Google Forms^®^, the data were stored in an Excel^®^ spreadsheet. Descriptive statistics were used to analyze participant data. The open-ended item asking respondents to identify at least one element in each SWOT category-Strengths, Weaknesses, Opportunities, and Threats-allowed them to respond freely using keywords, which were retained and used in constructing the SWOT matrix.

## RESULTS

Eleven health professionals participated in the study, including nurses, biochemists, and biomedical scientists. Although biologists were eligible to participate, none were included in the final sample because of the exclusion criteria. Among participants, 90.9% were female, with a mean age of 34.5 years. Time since graduation ranged from 3 to 21 years, with a mean of 9.5 years. Length of service at the institution ranged from 1 to 17 years. Table 1 presents the participants’ characteristics.

**Table 1:** Characteristics of study participants, Chapecó, Santa Catarina, Brazil, 2025

Profession	n	%
Nurse	5	45.4
Biochemist	3	27.3
Biomedical scientist	3	27.3
**Sex**		
Female	10	90.9
Male	1	9.1
**Graduate education**		
Graduate certificate		72.7
Academic graduate degree	3	27.3
**Weekly workload**		
40 hours	7	63.6
44 hours	3	27.3
30 hours	1	9.1
**Work shift**		
Mixed	10	90.9
Morning	1	9.1

Table 2 presents the factors considered most relevant because they pose the greatest challenges in day-to-day work related to transfusion safety and may directly affect the transfusion process. Regarding the implementation of a management tool designed to support transfusion safety, all participants (N = 11) reported that such a tool is needed.

**Table 2:** Most relevant factors interfering with a safe transfusion process, Chapecó, Santa Catarina, Brazil, 2025

Factor	n	%
Collection/identification of pretransfusion samples	10	90.9
Monitoring of the transfusion procedure	9	81.8
Team communication	8	72.7
Posttransfusion monitoring	7	63.6
Completion of the Transfusion Service Request Form	7	63.6
Blood component selection	7	63.6
Conduct of pretransfusion testing	6	54.5
Blood component transfusion	5	45.5
Recording of the pretransfusion sample	4	36.4
Recording of pretransfusion test results	3	27.3
Unit-to-patient matching	3	27.3

Table 3 presents the items that participants considered important for developing a transfusion safety checklist.

**Table 3:** Items identified as important for building a transfusion safety checklist according to the preanalytical, analytical, and postanalytical stages, Chapecó, Santa Catarina, Brazil, 2025

	Transfusion process stage	n	%
**Important elements for transfusion safety**			
Confirm that the TSRF^*^ includes the correct diagnosis and clinical indication.	Preanalytical	9	81.8
Confirm correct identification of the patient and pretransfusion sample (full name, date of birth, and medical record number).	Preanalytical	8	72.7
Confirm the type and quantity of blood components requested on the TSRF.	Preanalytical	6	54.5
Verify that the patient or legal representative has signed the Informed Consent Form.	Preanalytical	4	36.3
Confirm the transfusion order in the paper or electronic medical record.	Preanalytical	4	36.3
For emergency transfusion requests, verify that the transfusion authorization form has been completed, signed by the physician, and attached to the other documents.	Preanalytical	2	18.2
**Important elements for transfusion safety**			
Confirm that the information on the TSRF matches the collected pretransfusion sample.	Analytical	6	54.5
Perform a double-check before proceeding to the postanalytical stage.	Analytical	6	54.5
Select blood components according to the recipient’s diagnosis.	Analytical	5	45.5
Perform pretransfusion testing and record the results.	Analytical	4	36.3
**Important elements for transfusion safety**			
Monitor vital signs before transfusion, during the first 10 minutes, and at the end of the transfusion.	Postanalytical	8	72.7
Perform positive patient identification at the time of blood component transfusion (double-check).	Postanalytical	6	54.5
Fully document the transfusion in the medical record and on the TSRF (blood component number and type, vital signs, date, time, and responsible professional), as well as any incidents.	Postanalytical	5	45.5
Perform unit-to-patient matching by visually inspecting the unit, checking the transfusion card data against the request form, and signing the release field for the responsible professional.	Postanalytical	4	36.3
Record delivery to restricted-access units where transfusions are performed.	Postanalytical	4	36.3
Closely monitor for possible transfusion reactions and report adverse events.	Postanalytical	4	36.3

* *TSRF - Transfusion Service Request Form.*

When asked about transfusion care, seven professionals (63.6%) reported that this topic had not been addressed during their professional education. Regarding the support provided by their institution for education, training, and capacity-building in this area, seven professionals (63.6%) reported that such support was sometimes provided, whereas four participants (36.4%) reported that it was always provided. All participants reported taking courses or training programs and attending scientific events (such as seminars, symposia, or conferences) on the topic, in addition to reading publications in scientific journals.

For the SWOT analysis, participants were asked through the online questionnaire to identify the strengths, weaknesses, opportunities, and threats they perceived in relation to transfusion care within their service. Chart 1 summarizes the information compiled from the SWOT analysis.

**Chart 1:** Representation of the strengths, weaknesses, opportunities, and threats identified by health professionals involved in transfusion care, Chapecó, Santa Catarina, Brazil, 2025

STRENGHTS	WEAKNESSES
Skilled and committed team Continuing education Semiautomated processes for pretransfusion testing Professional expertise Coordination across sectors (supported by computerized systems) Standardized protocols Highly qualified professionals recognized as national references	Staffing constraints in hospital-based transfusion services High staff turnover Work overload Ineffective and/or insufficient training Lack of analysis of transfusion request data and individual review of each request
**OPPORTUNITIES**	**THREATS**
Implementation of new protocols to enhance transfusion safety Implementation of label printing for sample identification and patient wristbands Experience sharing across institutions Strengthening continuing education for technical and administrative teams throughout the network Closer collaboration with smaller services, creating opportunities for knowledge transfer	Reduced blood component inventory Working on autopilot and professional overconfidence Treating the transfusion procedure as routine or lack of commitment among the professionals who perform transfusions Shortage of qualified professionals

## DISCUSSION

The analysis highlights the need for strategic actions to strengthen professional recognition, support ongoing professional development, modernize processes, and strengthen safe practices across all levels of the blood center network. The opportunities identified in the SWOT analysis can significantly help mitigate weaknesses and address the identified threats, helping create a safer and more effective environment for blood component transfusions.

Most participants also indicated the need for a tool to support transfusion safety, structured around the three distinct yet interconnected stages of blood component transfusion: preanalytical, analytical, and postanalytical. The preanalytical stage includes pretransfusion sample collection and identification, completion of the TSRF, and documentation of the pretransfusion sample^(20,21)^. The transfusion process begins with the collection and identification of the pretransfusion sample, which nearly all participants identified as highly relevant to transfusion safety.

The preanalytical stage is critical to the success of the transfusion process because the most common external factors-such as distractions, simultaneous sample collection, failure to label the sample immediately, and communication breakdowns that can compromise sample collection and identification-can be managed by the professional collecting the sample, since the focus should be on safe sample collection, storage, and transport^(22)^. Professionals should adopt a proactive stance and communicate assertively with the team, the patient, and the family member, as the required patient identifiers must be verified at the time of collection. Some controllable variables, such as tourniquet pressure during venipuncture and collection technique, should be monitored and adjusted, as hemolyzed or clotted samples cannot be processed, requiring repeat collection and exposing patients to harm. Educational measures can help improve these processes and, in turn, lead to better results, fewer errors, and greater patient safety^(23,24)^.

The analytical stage includes pretransfusion testing, blood component selection, and documentation of pretransfusion test results. Most testing is performed through automated processes, allowing greater control, accuracy, sensitivity, and precision. However, even with automation, this stage still depends on human involvement, so analytical errors can occur, including sample mix-ups, pipetting errors, use of out-of-specification reagents, and utilization of uncalibrated and/or unvalidated equipment^(25,26)^. In this regard, more than half of the participants (63.6%) identified test result documentation and blood component selection as relevant factors in the transfusion process that may lead to incorrect result reporting.

Lastly, the postanalytical stage involves documentation of unit-to-patient matching, blood component transfusion, transfusion monitoring, and posttransfusion monitoring. In this regard, nine participants (81.8%) identified transfusion monitoring as a critical factor in the process, consistent with another study^(27)^ that reviewed the medical records of patients who received blood component transfusions and found that adherence to safe practices was more frequent in the preanalytical stage than in the postanalytical stage. In that study, shortcomings were identified in documentation related to vital sign checks, as well as in recording the unit number and the name of the professional responsible for the transfusion in the medical record.

Team communication was also identified as a relevant factor for transfusion safety (72.7%) because it cuts across all stages of the transfusion process, reinforcing the importance of the nurse’s role in transfusion practice and the need for training, capacity-building, and dissemination of information on best practices during transfusion procedures^(28)^.

The items identified by participants as most relevant are consistent with findings in the literature^(29-31)^ and are also supported by Brazilian legislation. Consolidation Ordinance No. 5, dated September 28, 2017^(6)^, which consolidates the rules governing health actions and services within the Brazilian Unified Health System (SUS), includes, in Annex IV, the technical regulation for hemotherapy procedures. This regulation covers aspects such as completion of the TSRF, the indication for emergency transfusion, sample identification and sample validity period, verification of recipient data through document cross-checking, blood component selection, visual inspection, vital sign monitoring, medical record documentation, and recipient monitoring after transfusion.

Participants actively contributed to developing the minimum framework for a transfusion safety checklist. This contribution, together with findings from the scientific literature and current legislation, supports the development of a first version of the proposed management tool.

### Study limitations

This study is limited by the small number of participants when considered in a national context and by the fact that all participants were drawn from a single blood center network. However, because the network involved in the study accounts for 99% of transfusion services in Santa Catarina, the findings may also reflect the reality of transfusion services in other Brazilian states, which could likewise benefit from these results.

### Contributions to nursing, health, or public policy

This study contributes to transfusion safety, including patient safety. It also highlights the importance of ongoing educational actions for health professionals. Increasing these professionals’ awareness of the issue may help drive changes in the institutional setting of units that perform blood component transfusions, with positive effects on recipients’ lives and lower additional costs for health services resulting from adverse events.

## FINAL CONSIDERATIONS

Health professionals’ knowledge of best practices in blood component transfusion is essential to ensuring safe and effective care. The findings also point to the importance of institutional support for professionals’ continuing education, as well as to improvements in internal processes, to minimize preventable adverse events and enhance transfusion safety.

The situational diagnosis identified the preanalytical stage as a strength and the postanalytical stage as a weakness. Tools that can improve process safety emerged as opportunities, whereas professional overconfidence emerged as a threat because it may lead to preventable events.

By highlighting professionals’ perceptions of both the positive and negative aspects of their work experiences related to transfusion safety in transfusion services, the study prompted reflection on day-to-day professional practice, as well as on compliance with legislation and best practices in blood component transfusion, reinforcing each professional’s commitment to the service and, above all, to recipients. These findings will guide the development of the checklist, which can make a significant contribution to improving the safety of blood component transfusions performed across the Santa Catarina blood center network.

## Data Availability

The research data are available within the article.
