# Supplementary material for: Transfusion safety in transfusion services: health professionals’ knowledge
Source: Rev Bras Enferm. 2026 Aug 21;79:e20250470. doi: 10.1590/0034-7167-2025-0470 (PMC13505560; doi:10.1590/0034-7167-2025-0470)
Supplement: Translation

# Translation: Segurança transfusional no serviço de hemoterapia: conhecimento dos profissionais de saúde

**DOI:** 10.1590/0034-7167-2025-0470pt

**Authors:** Maíra Cássia Borges de Oliveira, Rosana Amora Ascari, Everaldo José Schörner, Leticia de Lima Trindade, Daiana de Mattia

**Affiliations:** I Universidade do Estado de Santa Catarina. Chapecó, Santa Catarina, Brasil; II Universidade Federal de Santa Catarina. Florianópolis, Santa Catarina, Brasil

**Keywords:** Serviço de Hemoterapia, Transfusão de Sangue, Segurança Transfusional, Pessoal de Saúde, Enfermagem.

## Abstract

**Objetivos:**

identificar o conhecimento dos profissionais de saúde de uma hemorrede sobre as boas práticas na transfusão de hemocomponentes e estimular a discussão crítica visando construir uma estrutura mínima para um *checklist* de segurança transfusional.

**Métodos:**

estudo transversal, exploratório e descritivo com 11 profissionais que atuam nas oito agências transfusionais gerenciadas pelo Centro de Hematologia e Hemoterapia de Santa Catarina.

**Resultados:**

a coleta de dados ocorreu em julho de 2025 mediante questionário estruturado contendo perguntas sobre o perfil sociodemográfico dos profissionais e levantamento sobre a segurança transfusional na hemorrede catarinense.

**Considerações Finais:**

o conhecimento dos profissionais sobre as boas práticas na transfusão de hemocomponentes é essencial para garantir um atendimento seguro e eficaz. Ao ressaltar as percepções dos profissionais sobre suas vivências laborais em serviços hemoterápicos, o estudo possibilitou reflexões sobre a prática profissional cotidiana.

## INTRODUÇÃO

No Brasil, os hemocomponentes são produzidos com base na doação de sangue total ou por aférese, realizada por doador voluntário, conforme estabelecido em legislação nacional e em regulamentos técnicos do Ministério da Saúde. Toda doação de sangue deve ocorrer de forma voluntária e solidária, sem gratificação direta ou indireta e com garantia do anonimato do doador^(1)^.

A transfusão de hemocomponentes é um procedimento moderno que visa restabelecer os componentes sanguíneos em deficiência devido a perdas agudas ou crônicas, tais como em traumas, cirurgias de grande porte e doenças oncológicas e hematológicas^(2,3)^.

No tocante a hemocomponentes, o método de aférese consiste em um procedimento automatizado no qual o sangue total do doador é processado em um equipamento especializado para separar e coletar seletivamente componentes como plaquetas, plasma, hemácias e granulócitos, devolvendo os demais elementos ao doador. A aférese permite a obtenção de maiores volumes e maior pureza dos hemocomponentes em comparação à doação convencional do sangue total, além de proporcionar maior segurança ao doador e ao receptor, com menor exposição a múltiplos doadores e redução do risco de reações adversas^(4)^.

As técnicas atuais de processamento permitem a obtenção e o armazenamento de diferentes hemocomponentes sanguíneos em condições que preservam suas características terapêuticas. Isso possibilita que o receptor receba apenas os componentes necessários, em menor volume, reduzindo os riscos associados à exposição da terapêutica transfusional^(5)^. Assim, uma única doação pode beneficiar diversos pacientes de maneira mais segura e eficaz. Nesse sentido, exames de qualificação de doador são realizados em todas as doações para garantir a segurança do doador e do receptor; entre esses exames, incluem-se os testes para a identificação de agentes infecciosos transmissíveis pelo sangue, os testes imuno-hematológicos e a triagem para hemoglobina S, voltada à detecção de mutação genética associada à doença falciforme^(6,7)^.

As condições clínicas de cada receptor, e não somente a análise de exames laboratoriais, devem ser consideradas para a determinação da transfusão. Isso porque, apesar dos avanços na captação de doadores, triagem, processamento do sangue e segurança transfusional, essa terapêutica ainda envolve riscos inerentes ao próprio processo, como imunossupressão, aloimunização e eventos adversos evitáveis^(8)^.

Dessa forma, como o procedimento transfusional não está livre de riscos, deve-se optar por essa terapêutica apenas quando não houver outra opção de tratamento indicada; essa decisão deve ser compartilhada com o paciente e/ou seus familiares, a fim de discutir os riscos e sanar as possíveis dúvidas, sendo recomendada a transfusão quando seus benefícios se sobrepõem aos riscos^(1)^. Em contextos envolvendo, por exemplo, casos de anemia grave, hemorragia aguda ou choque hemorrágico, a transfusão de hemocomponentes revela benefícios imediatos necessários, o que inclui a restauração do transporte de oxigênio, estabilização hemodinâmica e redução da mortalidade. Apesar dos riscos relacionados a reações transfusionais, a adoção de medidas restritivas à transfusão com critérios bem definidos demonstra melhoria nos desfechos clínicos, com minimização de complicações e redução da permanência hospitalar^(9,10)^.

O conhecimento e as habilidades dos profissionais de saúde são essenciais ao desenvolvimento e qualificação dos procedimentos de transfusão de hemocomponentes segura para alcançar bons desfechos clínicos, principalmente no tocante aos treinamentos e capacitações das equipes, a fim de evitar que eventos adversos aconteçam^(11)^. A Resolução nº 709 de 2022^(12)^ do Conselho Federal de Enfermagem (Cofen) enfatiza que o profissional técnico de enfermagem e o enfermeiro devem estar devidamente capacitados para realizar procedimentos como coleta, armazenamento, controle de qualidade e assistência a doadores e pacientes, visando garantir uma assistência competente, resolutiva e segura^(12)^. Além dos profissionais de enfermagem, atuam nos serviços de hemoterapia médicos, farmacêuticos, biomédicos e biólogos, responsáveis pela análise e testes pré-transfusionais, seleção de hemocomponentes conforme a necessidade de cada receptor, liberação de resultados, bem como resolução de discrepâncias^(13)^.

No estado de Santa Catarina (SC), as ações referentes ao ciclo do sangue são coordenadas pelo Centro de Hematologia e Hemoterapia de Santa Catarina (HEMOSC), responsável por 99% da cobertura estadual em hemoterapia, o que inclui as ações de captação e doação voluntária de sangue e medula óssea. A instituição garante controle e qualidade nas coletas, na qualificação do doador, produção e controle de qualidade dos hemocomponentes, bem como estocagem e distribuição para os serviços públicos e privados, realizando a gestão estadual do estoque de sangue^(14,15)^.

Diante do exposto, este estudo se justifica pela necessidade de identificar os conhecimentos dos profissionais de saúde sobre as boas práticas na transfusão de hemocomponentes, em consonância com a segurança transfusional. Futuramente, poderá subsidiar a construção de uma tecnologia gerencial que auxilie os profissionais de saúde atuantes em hemoterapia no processo de segurança transfusional. Acredita-se que a tecnologia em forma de *checklist* contribuirá com a segurança do paciente e qualidade da assistência prestada, para minimizar eventos adversos e contribuir com os Objetivos de Desenvolvimento Sustentável (ODS), especialmente o ODS 3 (Saúde e Bem-estar), ao promover cuidados seguros e de qualidade; o ODS 4 (Educação de Qualidade), ao fomentar a capacitação profissional; e o ODS 16 (Paz, Justiça e Instituições Eficazes), ao fortalecer a gestão ética e responsável nos serviços de saúde^(16)^. A pesquisa faz parte de um macroprojeto para a construção de uma tecnologia gerencial do tipo *checklist* a ser implantada no sistema da hemorrede catarinense. Ela aborda a primeira de quatro etapas propostas, representada pelo diagnóstico da realidade acerca do serviço de saúde em questão.

## OBJETIVOS

Identificar o conhecimento dos profissionais de saúde de uma hemorrede sobre as boas práticas na transfusão de hemocomponentes e estimular a discussão crítica visando construir uma estrutura mínima para um *checklist* de segurança transfusional.

## MÉTODOS

### Desenho do estudo

Trata-se de um estudo transversal, exploratório e descritivo de abordagem quantitativa, conforme os critérios STROBE^(17)^. Foi realizado em julho de 2025.

### Contexto

O cenário de estudo contemplou as oito agências transfusionais (AT) gerenciadas pelo Centro de Hematologia e Hemoterapia de Santa Catarina (HEMOSC), localizadas de forma distribuída no estado de Santa Catarina: Hospital Regional de São José (São José), Hospital Governador Celso Ramos (Florianópolis), Hospital Florianópolis (Florianópolis) e Hospital Infantil Joana de Gusmão (Florianópolis), Hospital Regional do Oeste (Chapecó), Hospital Hans Dieter Schmidt (Joinville), Maternidade Tereza Ramos (Lages) e Hospital Waldomiro Colautti (Ibirama)^(15)^.

O estudo foi conduzido de acordo com as diretrizes éticas estabelecidas pelos órgãos reguladores nacionais e internacionais e foi aprovado pelo Comitê de Ética em Pesquisa da Universidade do Estado de Santa Catarina e da Secretaria do Estado de Saúde de Santa Catarina (SES). Todos os participantes assinaram o Termo de Consentimento Livre e Esclarecido (TCLE).

A abordagem dos participantes ocorreu por meio de convite via e-mail à Coordenação Assistencial da Hemorrede. Nele constava a carta de apresentação a ser encaminhada aos possíveis participantes, informando os objetivos do estudo e as devidas orientações sobre sua participação, incluindo o link de acesso ao TCLE e, após o consentimento em ambiente virtual, a liberação do instrumento de coleta de dados. Foi estabelecido o prazo de 15 dias para retorno do possível participante, com envio de lembrete semanal para prorrogação do prazo, sem exceder dois lembretes. Nos casos de ausência de retorno por parte do possível participante, o convite era enviado ao profissional seguinte.

### Participantes

A amostra foi selecionada intencionalmente e composta por profissionais com ensino superior completo - enfermeiros, biomédicos, bioquímicos e biólogos - atuantes em AT da hemorrede catarinense. Foram excluídos os profissionais que estavam em afastamento, licença ou férias.

### Fonte de dados

O instrumento de coleta utilizado foi composto por dois blocos. O primeiro continha os dados sociodemográficos e experiência profissional dos participantes; e o segundo direcionava-se à segurança transfusional na hemorrede catarinense. Tal instrumento contava com 20 questões, algumas abertas e outras fechadas, elaboradas pelos pesquisadores com base na análise da literatura e da prática profissional. Foi disponibilizado aos participantes para pesquisa on-line, via *Google Forms^®^*.

Ainda, considerando o intuito da construção da tecnologia gerencial, o mesmo questionário aplicado aos participantes contemplou questões voltadas à matriz SWOT - *Strengths* (Pontos fortes), *Weaknesses* (Pontos fracos), *Opportunities* (Oportunidades) e *Threats* (Ameaças) - para identificar pontos fortes que precisam ser mantidos e pontos fracos que necessitam ser trabalhados^(18,19)^. Nessa direção, a referida matriz é uma ferramenta de gestão amplamente utilizada como base para planejamentos estratégicos e de gestão de organizações.

### Tamanho do estudo

A amostra elegível foi de 16 profissionais, levando em conta que cada AT é composta por 2 profissionais de ensino superior, sendo oito agências transfusionais na hemorrede catarinense.

### Métodos estatísticos

Após a coleta de dados via *Google Forms* ^®^, estes foram armazenados em uma planilha *Excel* ^®^. Foi utilizada estatística descritiva para analisar as informações dos participantes. A questão aberta para identificação de pelo menos um elemento referente aos aspectos de Pontos fortes, Pontos fracos, Oportunidades e Ameaças permitiu ao participante expressar-se livremente por meio de palavras chaves, as quais foram respeitadas na composição da matriz SWOT.

## RESULTADOS

Participaram da pesquisa 11 profissionais de saúde, entre eles enfermeiros, bioquímicos e biomédicos. Embora houvesse biólogos elegíveis para participar da pesquisa, não houve representação, considerando os critérios de exclusão. A caracterização dos participantes mostra que 90,9% dos profissionais são do sexo feminino, com idade média de 34,5 anos. O tempo de formação acadêmica variou de 3 a 21 anos, e a média de tempo de formação foi de 9,5 anos. O tempo de atuação na instituição variou de 1 a 17 anos. Na Tabela 1, a caracterização dos participantes é apresentada.

**Tabela 1:** Caracterização dos participantes do estudo, Chapecó, Santa Catarina, Brasil, 2025

Profissão	n	%
Enfermeiro	5	45,4
Bioquímico	3	27,3
Biomédico	3	27,3
**Sexo**		
Feminino	10	90,9
Masculino	1	9,1
**Especialização**		
Lato sensu	8	72,7
Stricto sensu	3	27,3
**Carga horária semanal**		
40 horas	7	63,6
44 horas	3	27,3
30 horas	1	9,1
**Turno de trabalho**		
Misto	10	90,9
Manhã	1	9,1

A Tabela 2 apresenta os fatores de maior relevância que trazem dificuldades no processo de trabalho diário relacionado à segurança transfusional e que podem interferir diretamente no processo transfusional. No que diz respeito à implementação de um instrumento de tecnologia gerencial voltado à segurança transfusional, todos os participantes (N = 11) responderam que é necessário ter um instrumento de suporte à segurança transfusional.

**Tabela 2:** Fatores de maior relevância que interferem no processo transfusional seguro, Chapecó, Santa Catarina, Brasil, 2025

Fator	n	%
Coleta/identificação de amostras pré-transfusionais	10	90,9
Monitoramento do ato transfusional	9	81,8
Comunicação entre a equipe	8	72,7
Monitoramento pós-transfusional	7	63,6
Registro da Solicitação de Serviço Hemoterápico	7	63,6
Seleção de hemocomponentes	7	63,6
Execução dos exames pré-transfusionais	6	54,5
Transfusão de hemocomponentes	5	45,5
Registro da amostra pré-transfusional	4	36,4
Registro de Resultados de Exames Pré-transfusionais	3	27,3
Vínculo entre bolsa do hemocomponente e receptor	3	27,3

A Tabela 3 apresenta os elementos que os participantes julgam importantes para a composição de um *checklist* de segurança transfusional.

**Tabela 3:** Elementos sinalizados como importantes para a construção de um *checklist* de segurança transfusional seguindo as etapas pré-analítica, analítica e pós-analítica, Chapecó, Santa Catarina, Brasil, 2025

	Etapa do processo transfusional	n	%
**Elementos importantes para a segurança transfusional**			
Conferir a SSH^*^ com diagnóstico e indicação clínica preenchidos corretamente.	Pré-analítica	9	81,8
Conferir a identificação correta do paciente e da amostra pré-transfusional (nome completo, data de nascimento e número de prontuário).	Pré-analítica	8	72,7
Conferir, na SSH, as informações sobre tipo e quantidade de hemocomponentes solicitados.	Pré-analítica	6	54,5
Verificar se o Termo de Consentimento Esclarecido foi assinado pelo paciente ou responsável.	Pré-analítica	4	36,3
Conferir a prescrição de transfusão no prontuário físico ou eletrônico.	Pré-analítica	4	36,3
Em caso de solicitação de transfusão de emergência, conferir se o termo de autorização de transfusão foi preenchido, assinado pelo médico e anexado aos demais documentos.	Pré-analítica	2	18,2
**Elementos importantes para a segurança transfusional**			
Conferir se os dados da SSH e da amostra pré‑transfusional coletada são iguais.	Analítica	6	54,5
Realizar a dupla conferência antes de passar à fase pós-analítica.	Analítica	6	54,5
Selecionar os hemocomponentes conforme o diagnóstico do receptor.	Analítica	5	45,5
Realizar os testes pré-transfusionais e registrar os resultados obtidos.	Analítica	4	36,3
**Elementos importantes para a segurança transfusional**			
Monitorar os sinais vitais antes da transfusão, nos primeiros 10 minutos e ao final da transfusão.	Pós-Analítica	8	72,7
Conferir a identificação ativa do paciente no momento da transfusão do hemocomponente (dupla checagem).	Pós-analítica	6	54,5
Registrar integralmente a transfusão no prontuário e na solicitação (número e tipo do hemocomponente, sinais vitais, data, hora e profissional), bem como eventuais intercorrências.	Pós-analítica	5	45,5
Realizar o vínculo entre a bolsa e o paciente, fazendo a inspeção visual, conferindo os dados do cartão transfusional com a solicitação e assinando no campo do responsável pela liberação.	Pós-analítica	4	36,3
Registrar a entrega para setores fechados que realizem o ato transfusional.	Pós-analítica	4	36,3
Observar rigorosamente possíveis reações transfusionais e notificar eventos adversos.	Pós-analítica	4	36,3

* *SSH - Solicitação de Serviço Hemoterápico.*

Quando questionados sobre o tema “Atenção em saúde na hemoterapia”, sete profissionais (63,6%) relataram que esse tema não havia sido abordado durante sua formação profissional. Quanto ao incentivo oferecido pela instituição em que atuavam para a realização de formações, capacitações e treinamentos sobre o tema, sete profissionais (63,6%) responderam que esse incentivo ocorre às vezes, enquanto quatro participantes (36,4%) apontaram que ele ocorre sempre. Todos os participantes referem participar de cursos, capacitações ou eventos científicos (como jornadas, simpósios ou congressos) sobre a temática, além de ler publicações da área em revistas científicas.

No que tange à construção da matriz SWOT, os participantes foram instigados a responder, por meio do questionário on-line, quais pontos fortes, pontos fracos, oportunidades e ameaças eles identificavam em relação ao atendimento hemoterápico do serviço no qual atuavam. O Quadro 1 apresenta o compilado de informações resultantes da matriz SWOT.

**Quadro 1:** Representação dos pontos fortes, pontos fracos, oportunidades e ameaças identificados pelos profissionais de saúde do atendimento hemoterápico, Chapecó, Santa Catarina, Brasil, 2025

PONTOS FORTES	PONTOS FRACOS
Equipe capacitada e comprometida Capacitação contínua Processos semiautomatizados para a realização de testes pré-transfusionais Qualificação dos profissionais Capacidade de articulação entre setores (uso de sistemas informatizados) Protocolos padronizados Profissionais altamente capacitados e considerados referências nacionais	Limitações no quadro de pessoal das agências transfusionais Alta rotatividade de colaboradores Sobrecarga profissional Capacitações não efetivas e/ou insuficientes Ausência de tratamento dos dados dos pedidos de transfusão e de avaliação individual de cada pedido
**OPORTUNIDADES**	**AMEAÇAS**
Implantação de novos protocolos para aumentar a segurança transfusional Implementação de etiquetadora para identificação de amostras e pulseiras de pacientes Compartilhamento de experiências entre instituições Fortalecimento da capacitação contínua das equipes técnicas e administrativas em todos os pontos da rede Aproximação com serviços menores, com possibilidade de transferência de conhecimento	Estoque reduzido de hemocomponentes Atuação em modo automático e excesso de confiança Banalização do ato transfusional ou falta de comprometimento por parte dos profissionais que realizam as transfusões Escassez de profissionais qualificados

## DISCUSSÃO

A análise evidencia a necessidade de ações estratégicas voltadas à valorização profissional, qualificação contínua, modernização dos processos e fortalecimento das práticas seguras em todos os níveis da hemorrede. O aproveitamento das oportunidades mapeadas na matriz SWOT pode contribuir significativamente para mitigar os pontos fracos e enfrentar as ameaças identificadas, promovendo um ambiente mais seguro e eficaz para a realização das transfusões de hemocomponentes.

Ainda, a maioria dos participantes aponta para a necessidade de um instrumento que auxilie na segurança transfusional, estruturado segundo as três etapas distintas, mas interligadas, da transfusão de hemocomponentes: pré-analítica, analítica e pós-analítica. A etapa pré-analítica contempla o momento da coleta e identificação de amostras pré-transfusionais, registro da solicitação do hemocomponente e registro da amostra pré-transfusional^(20,21)^. O processo transfusional tem início com a coleta e identificação da amostra pré-transfusional, aspecto apontado pela quase totalidade dos participantes como de grande relevância para a segurança transfusional.

A fase pré-analítica é crucial para o sucesso da transfusão, pois os fatores externos mais comuns - como distrações, coleta de amostras simultâneas, ausência de identificação imediata e falhas na comunicação, que podem interferir na coleta e identificação da amostra - são passíveis de manejo pelo próprio coletador, uma vez que o foco deve ser a segurança na coleta, armazenamento e transporte da amostra^(22)^. O profissional deve adotar uma postura proativa e comunicação assertiva, tanto com a equipe quanto com o paciente e seu familiar, já que os itens de identificação segura do paciente deverão ser verificados no ato da coleta. Algumas variáveis controláveis, como a pressão do torniquete para realizar a punção venosa e a forma da coleta, devem ser supervisionadas e ajustadas, porque amostras hemolisadas e coaguladas não pode ser processadas, exigindo novas coletas e expondo o paciente a prejuízos. Medidas educativas tendem a ser efetivas para ajustes nos processos e, consequentemente, auxílio na obtenção de melhores resultados, com menos erros e maior segurança para os pacientes^(23,24)^.

A etapa analítica contempla as atividades de execução dos exames pré-transfusionais, seleção do hemocomponente e registro de resultado dos exames pré-transfusionais. A execução dos testes, em sua grande maioria, se dá por meio de processo automatizado, permitindo maior controle, acurácia, sensibilidade e precisão. Entretanto, mesmo com a automatização, este ainda é um processo dependente de participação humana, de modo que erros analíticos podem ocorrer, tais como troca de amostras, erro de pipetagem, uso de reagentes fora do padrão estabelecido e utilização de equipamentos não calibrados e/ou não validados^(25,26)^. Nesse sentido, mais da metade dos participantes (63,6%) apontou que o registro de resultados e seleção do hemocomponente são fatores relevantes no processo transfusional que podem levar à emissão de resultados incorretos.

Por fim, a etapa pós-analítica diz respeito ao registro do vínculo entre hemocomponente e receptor, transfusão do hemocomponente, monitoramento transfusional e monitoramento pós transfusional. Nesse sentido, nove participantes (81,8%) sinalizaram que o monitoramento transfusional é um fator crítico no processo, convergindo com outro estudo^(27)^, que analisou prontuários de pacientes submetidos à transfusão de hemocomponentes e apontou que a adesão às práticas seguras ocorre com maior frequência na etapa pré-analítica do que na pós-analítica. Nesse contexto, foram evidenciadas falhas no preenchimento de registros relacionados à verificação de sinais vitais, bem como no registro em prontuário do número da bolsa e do nome do responsável pela transfusão.

Ainda, a comunicação entre a equipe foi sinalizada como fator relevante para a segurança transfusional (72,7%), pois permeia todas as etapas transfusionais, o que reforça a importância da atuação do enfermeiro na prática transfusional e a necessidade de treinamentos, capacitações e disseminação de informações sobre as boas práticas no ato transfusional^(28)^.

Os itens sinalizados como de maior relevância pelos participantes corroboram achados na literatura^(29-31)^ e também encontram respaldo na legislação brasileira. A Portaria de Consolidação nº 5, de 28 de setembro de 2017^(6)^, que consolida as normas sobre as ações e os serviços de saúde do Sistema Único de Saúde (SUS), traz, no anexo IV, o regulamento técnico de procedimentos hemoterápicos. Esse regulamento contempla aspectos como o preenchimento da SSH, a indicação de transfusão de emergência, a identificação das amostras e o prazo de validade das amostras, a conferência dos dados do receptor com o confronto de documentos, a seleção de hemocomponentes, a inspeção visual, a verificação de sinais vitais, os registros em prontuário e a vigilância do receptor após a transfusão.

Os participantes contribuíram ativamente para o desenvolvimento de uma estrutura mínima de um *checklist* relacionado à segurança transfusional. Essa contribuição em conjunto com os achados da literatura científica e legislação vigente permitem a construção de uma primeira versão da tecnologia proposta.

### Limitações do estudo

As limitações deste estudo podem estar relacionadas ao pequeno número de participantes em âmbito nacional e o fato de os participantes estarem vinculados a uma única hemorrede. Contudo, pela robustez da hemorrede envolvida no estudo, representando 99% dos serviços hemoterápicos de Santa Catarina, os achados deste estudo podem se assemelhar a serviços hemoterápicos de outros estados brasileiros, que também se beneficiariam desses resultados.

### Contribuições para as áreas de enfermagem, saúde ou políticas públicas

O estudo traz contribuições para a segurança transfusional, que inclui a segurança do paciente. Além disso, ressalta a importância de ações educativas contínuas voltadas aos profissionais de saúde. A sensibilização desses profissionais para a temática pode favorecer mudanças no cenário institucional das unidades que realizam transfusão de hemocomponentes, com impactos positivos na vida dos receptores e redução de custos adicionais nos serviços de saúde decorrentes de eventos adversos.

## CONSIDERAÇÕES FINAIS

O conhecimento dos profissionais de saúde sobre as boas práticas na transfusão de hemocomponentes é essencial para garantir um atendimento seguro e eficaz. Evidencia-se apoio institucional com a formação contínua dos profissionais, bem como a importância da qualificação dos processos internos, visando minimizar eventos adversos evitáveis e ampliar os níveis de segurança hemoterápica.

O diagnóstico da realidade possibilita compreender a etapa transfusional pré-analítica como ponto forte e a etapa pós-analítica como ponto fraco. Ferramentas capazes de aumentar a segurança do processo mostram-se como oportunidades, enquanto o excesso de confiança dos profissionais se destaca como ameaça por poder levar à ocorrência de eventos evitáveis.

Ao ressaltar as percepções sobre suas vivências laborais relacionadas à segurança transfusional em serviços hemoterápicos, tanto positivas quanto negativas, o estudo possibilitou a reflexão sobre a prática profissional cotidiana bem como o cumprimento da legislação e das boas práticas em transfusão de hemocomponentes, reforçando o compromisso de cada profissional com o serviço e, sobretudo, com os receptores. Esse conhecimento orientará o desenvolvimento do *checklist,* que pode contribuir significativamente para o aumento da segurança das transfusões de hemocomponentes realizadas na hemorrede catarinense.

## Data Availability

Os dados de pesquisa estão disponíveis no corpo do artigo.
